# Entomological impact of indoor residual spraying with pirimiphos-methyl: a pilot study in an area of low malaria transmission in Senegal

**DOI:** 10.1186/s12936-018-2212-x

**Published:** 2018-02-05

**Authors:** Ousmane Sy, El Hadji Amadou Niang, Magatte Ndiaye, Lassana Konaté, Abdoulaye Diallo, Elhadji Conco Ciré Ba, Fassiath Tairou, Elhadji Diouf, Badara Cissé, Oumar Gaye, Ousmane Faye

**Affiliations:** 1 Laboratoire d’Ecologie Vectorielle et Parasitaire, Faculté des Sciences et Techniques, Université Cheikh Anta Diop, Dakar, Senegal; 2 Laboratoire de Parasitologie Médicale, Faculté de Médecine, Pharmacie et d’Odonto-stomatologie, Université Cheikh Anta Diop, Dakar, Senegal; 3 Institut de Recherche pour le Développement (IRD), Dakar, Senegal; 4 London School of Hygiene & Tropical Medicine, London, UK

**Keywords:** Hotspot, IRS, Pirimiphos-methyl, Malaria, Elimination, Senegal

## Abstract

**Background:**

Scaling-up of effective anti-malarial control strategies in Central-West region of Senegal has resulted in the sharp decline in malaria prevalence in this area. However, despite these strategies, residual malaria transmission has been observed in some villages (hot spots). The objective of this study was to assess the impact of indoor residual spraying (IRS) with pirimiphos-methyl on malaria transmission in hot spot areas.

**Methods:**

The malaria vector population dynamics were monitored in each of the six selected villages (4 of which used IRS, 2 were unsprayed control areas) using overnight human landing catches (HLC) and pyrethrum spray catches (PSC). The host source of blood meals from freshly fed females collected using PSC was identified using the direct ELISA method. Females caught through HLC were tested by ELISA for the detection of *Plasmodium falciparum* circumsporozoite protein and *Anopheles gambiae* complex was identified using PCR.

**Results:**

Preliminary data shown that the densities of *Anopheles* populations were significantly lower in the sprayed areas (179/702) compared to the control. Overall, malaria transmission risk was 14 times lower in the intervention zone (0.94) compared to the control zone (12.7). In the control areas, three *Anopheles* species belonging to the Gambiae complex (*Anopheles arabiensis*, *Anopheles coluzzii* and *Anopheles melas*) maintained the transmission, while only *An*. *coluzzii* was infective in the sprayed areas.

**Conclusion:**

The preliminary data from this pilot study showed that IRS with the CS formulation of pirimiphos-methyl is likely very effective in reducing malaria transmission risk. However, additional studies including further longitudinal entomological surveys as well as ecological and ethological and genetical characterization of vectors species and their populations are needed to better characterize the entomological impact of indoor residual spraying with pirimiphos-methyl in the residual transmission areas of Senegal.

## Background

As in many other countries of the WHO Africa Region, a drastic decline in the global malaria burden over 15 years has been recorded in Senegal because of the integration and the scale-up of effective malaria control strategies. A subnational decrease in morbidity and mortality has been recorded in several countries and territories with on-going malaria transmission. The 2015 WHO Malaria Report reported that the estimation of case incidence rate fell by at least 75% between 2000 and 2015 in three countries of the African Region, including Senegal. This progress was made possible through the roll-out of effective prevention strategies, with long-lasting insecticide-treated nets (LLINs) and indoor residual spraying (IRS) being the core interventions [1]. These two measures account for almost 60% of global investment in malaria control [2].

The first pilot study of IRS as a wide-scale intervention was set up in the three health districts of Richard Toll, Nioro, and Velingara, after Senegal received the support of the US President’s Malaria Initiative (PMI) project in 2007. Overall, 76,279 structures housing around 700,000 people were sprayed [3, 4].

In addition to LLINs and IRS, between 2008 and 2010 four health districts located in the administrative departments of Fatick, Mbour, Niakhar and Bambey were selected by the Parasitology Department of the Université Cheikh Anta Diop of Dakar in collaboration with the National Malaria Control Programme (NMCP) to implement the roll-out of a pilot chemoprevention strategy named Seasonal Malaria Chemo-prevention (SMC) [5]. The SMC strategy involves administration of a single dose of sulfadoxine-pyrimethamine and amodiaquine to children under 10 during the peak malaria transmission season.

The overall control interventions have resulted in the nation-wide decrease of the malaria burden, most notably in the central west areas of Senegal [5]. As a result of this, malaria is mainly limited to areas known as “hot spots”, where transmission is consistently higher than the country’s average. Even in these hot spot areas of residual transmission, malaria incidence is still relatively low. To try and eliminate malaria from the hot spots in the four health districts of the Central West region of Senegal, it was decided to implement two successive rounds of targeted IRS interventions as part of a pilot study from the 5th to the 24th August then from the 18th July to the 27th August respectively during the rainy seasons in 2013 and in 2014, with the main objective of testing two rounds of targeted IRS-based vector control.

This paper reports the main results of this pilot study, focusing on the entomological impact of pirimiphos-methyl (ACTELLIC 300 CS, 0-2-diethylamino-6-methylpyrimidin-4-yl 0, 0-dimethylphosphorothioate) targeted IRS intervention in these areas of residual malaria transmission in Senegal.

## Methods

### Study area and sites

**Fig. 1:**
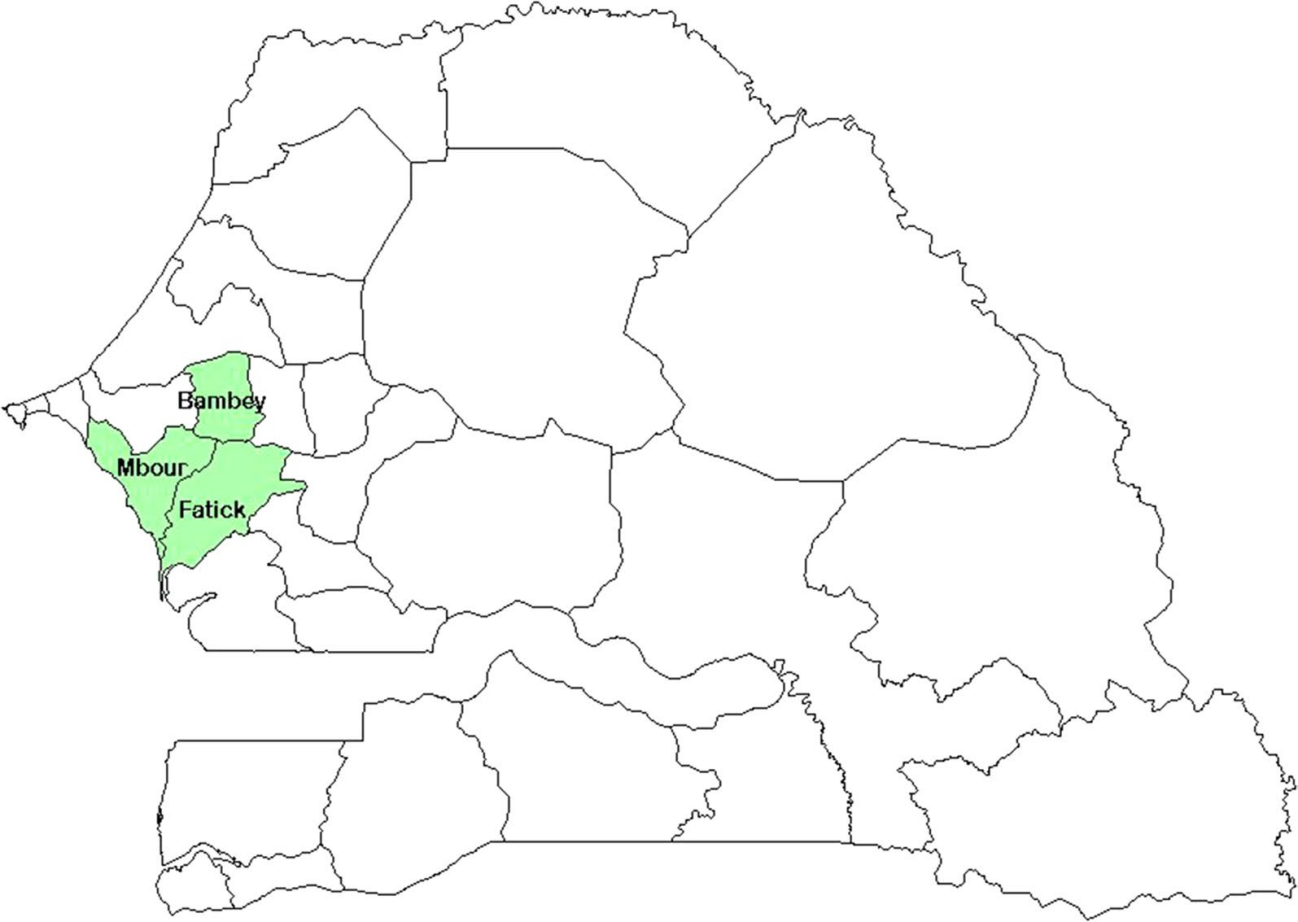
Location of the study health districts (Mbour, Fatick and Bambey)

The study area is located in the Central-West region of Senegal overlapping the administrative departments of Fatick, Mbour, Niakhar and Bambey (Fig. 1). It belongs to the Sahelo-Sudan bio-geographic Domain, characterized by the variability of rainfall lasting from June to October. The average rainfall for the last 2 years varied between 400 and 600 mm [6] and the mean temperature was 28 °C with high thermal amplitude.

For this study, villages were stratified by their malaria transmission level using previous data on the nationwide malaria incidences. An area was considered as a hot spot if 6 or more native inhabitants had confirmed malaria diagnoses reported during the previous year. The study took place in six rural communities, with the higher malaria transmission rates (hot spots) and representative of main ecological characteristics of the study area. Located, in the so-called Saloum ecological region, the study area is mainly characterized by degraded shrub and tree savannas altered by open agricultural parkland. Farming activities such as food (maize, millet) and cash crops (peanuts) are the economics activities of the local populations. Trade and breeding of domestic animals (cows, sheep, goats, and chicken), which are parked around human dwellings, are also common practices. Houses are of traditional types with mud walls and thatched or corrugated iron roofs. Lined with the National Malaria Control Programme (NMCP) recommendations for better-targeted vector control (VC) interventions, two IRS sprayed rounds were initially scheduled with the pirimiphos-methyl (ACTELLIC 300 CS) at the beginning and at the middle of the raining season to cover the transmission season in the host-spots. However, only one spray round was implemented in some sites, due to the lack of financial resource.

Taking into account the above situation, the localities of Toucar (14°32.189′; 16°28.497′) and Djilakh (14°30.989′; 16°52.009′) with two spray rounds, and the villages of Gate Diocoul 2 (14°31.842′; 16°31.789′), Takhoum Ndoundour (14°28.979′; 16°53.657′) and Keur Massouka (14°33′687′′; 16°55′722′′) sprayed once were selected to monitor the dynamics of the vectors population and malaria transmission in the study following the IRS campaign. The Toucar (14°32.189′; 16°28.497′) and Djilakh (14°30.989′; 16°52.009′) in the districts of Niakhar and Mbour were sprayed twice. The unsprayed sites of Gate Diocoul 2 (14°31.842′; 16°31.789′) and Keur Martin (14°24.511′;16°34.275′) presenting similar eco-epidemiological characteristic and located next to the IRS project zone were chosen as the negative control arm of this pilot study of the entomological impact of IRS with the ACTELLIC 300 CS.

### Mosquito sampling and processing

The malaria vector populations dynamics was monitored in each of the six selected villages (4 sprayed and 2 unsprayed controls) using overnight human landing catches (HLC) during two successive nights per month (both inside and outside three houses per village) and pyrethrum spray catches (PSC) early in the morning in ten randomly selected rooms per village. In both communities (IRS and unsprayed), human dwellings are mainly of traditional types with mud walls and thatched or corrugated iron roofs. Therefore, the sampling of HLC and PSC rooms was done randomly to be representative of as much as possible of ecological differences in each study villages.

Upon collection, mosquitoes were morphologically identified to genus level, and anophelines were subsequently identified to species level using morphological keys [7]. For each collection, 30% (N > 30 specimens) to 100% (N < 30 specimens) randomly sampled females of *Anopheles gambiae* sensu lato (s.l.) caught on human were dissected to determine the parity rate. The blood meals from freshly fed females collected using PSC were squashed onto Whatman filter paper and dried for host source identification. All the mosquito samples collected were stored individually in numbered vials with desiccant until laboratory processing.

The origin of blood meals was identified using the direct ELISA method from Beier et al. [8]. The heads and thoraces of host-seeking females were tested by ELISA for the detection of *Plasmodium falciparum* circumsporozoite protein (CSP) using the procedure of Wirtz et al. [9]. Members of the *An. gambiae* complex were identified by the PCR method described by Wilkins et al. [10].

### Data analysis

#### Measured parameters

The human-biting rate (HBR) was calculated for each species collected by HLC as the ratio of the total number of captured specimens to the total person-nights for the collection period. The parity rate was estimated as the proportion of parous over the total dissected. The indoor resting density was defined as the number of mosquitoes per room collected by PSC. The circumsporozoite rate was calculated as the proportion of the total number of mosquitoes infected with *P. falciparum*. The anthropophilic rate was calculated as the proportion of females with human blood out of the total tested. The entomological inoculation rate (EIR) was calculated from the result of the human-biting rate (HBR) and the CSP rate of mosquitoes collected from night catches.

#### Statistical analysis

All the measured parameters were computed and analysed using the free software R-gui 2.15.1 version. Data were compared with the Pearson chi^2^ or Fisher exact tests where applicable with the statistical significant threshold set at P value ≤ 0.05.

## Results

### Mosquito densities and species composition

A total of 38,039 mosquitoes of three genera (37,129 *Culex*, 208 *Aedes* and 702 *Anopheles*) were collected from October 2013 to April 2015 using both collection methods. Of this total, 62.7% were captured in the unsprayed control areas. During the study period, malaria vector densities were significantly different between the treated and control villages (Table 1) whatever collection method was considered (Treated: HLC = 77, PSC = 102; Control: HLC = 365, PSC = 140). Roughly 74.5% (523/702) of anopheline species were captured in the unsprayed control areas (χ2  =  335.18, df = 1, P  <  0.001). *Anopheles gambiae* s.l. was the predominant species (n = 684) among the *Anopheles* genus; the only other anopheline species encountered in the study area was *Anopheles pharoensis,* with all the eighteen specimens collected in the control locality of Keur Martin (health district of Fatick).

**Table 1:** Seasonal variation of *Anopheles gambiae* s.l. density in the study area

Month-year	Control				Sprayed once				Sprayed twice			
	Gate Diocoul 2		Keur Martin		Takhoum Ndoundour		Keur Massouka		Toucar centre		Djilakh	
	Collected	IRD	Collected	IRD	Collected	IRD	Collected	IRD	Collected	IRD	Collected	IRD
Oct-2013	0	0	13	1.3	0	0	9	0.9	7	0.7	4	0.4
Dec-2013	0	0	35	3.5	0	0	0	0	2	0.2	0	0
Mar-2014	0	0	0	0	0	0	0	0	0	0	0	0
Sep-2014	0	0	42	4.2	0	0	43	4.3	12	1.2	3	0.3
Oct-2014	0	0	45	4.5	0	0	0	0	7	0.7	13	1.3
Dec-2014	0	0	2	0.2	0	0	0	0	0	0	0	0
Mar-2015	0	0	1	0.1	0	0	0	0	2	0.2	0	0
Apr-2015	0	0	2	0.2	0	0	0	0	0	0	0	0
Total	0	0	140	1.75	0	0	52	0.65	30	0.37	20	0.25

*IRD* indoor resting density

### Biting and resting behaviours of *Anopheles gambiae* s.l. population

The mean number of bites per person per night (bpn) varied significantly for *An. gambiae* s.l. females between the two areas, with the highest aggressiveness observed in the unsprayed control villages (1.9 bpn) compared to the sprayed areas (villages with one spray round: 0.067 bpn; in villages with two spray rounds: 0.33 bpn). However, biting occurred more frequently outdoors in both the treated (53.2%) and the unsprayed control areas (61%) (Table 2).

**Table 2:** Seasonal variation of human biting rates and endophagy rates of *An. gambiae* *s.l*

Month-year	Control						Sprayed once						Sprayed twice					
	Gate Diocoul 2			Keur Martin			Takhoum Ndoundour			Keur Massouka			Toucar centre			Djilakh		
	Caught	HBR	Indoor	Caught	HBR	Indoor (%)	Caught	HBR	Indoor (%)	Caught	HBR	Indoor (%)	Caught	HBR	Indoor (%)	Caught	HBR	Indoor (%)
Oct-2013	0	0	–	32	2.66	16.60	1	0.08	100	0	0	–	4	0.33	50	13	1.08	30.60
Dec-2013	0	0	–	72	6	38.8	0	0	–	0	0	–	5	0.42	40	0	0	–
Mar-2014	0	0	–	1	0.08	0	0	0	–	0	0	–	0	0	–	0	0	–
Sep-2014	0	0	–	165	13.75	35.10	1	0.08	100	7	0.58	14.20	2	0.17	50	35	2.92	54.20
Oct-2014	1	0.08	0	92	7.6	53.20	2	0.17	50	0	0	–	1	0.08	100	4	0.33	50
Dec-2014	0	0	–	2	0.16	50	0	0	–	0	0	–	0	0	–	0	0	–
Mar-2015	0	0	–	0	0	–	0	0	–	0	0	–	0	0	–	0	0	–
Apr-2015	0	0	–	0	0	–	1	0.08	0	1	0.08	100	0	0	–	0	0	–
Total	1	0.01	0	364	3.79	39	5	0.05	60	8	0.08	25	12	0.12	50	52	0.54	48

*HBR* human biting rates, *Indoor* Indoor biting rates

During the study period, the resting density of *An. gambiae* s.l. females was significantly different between treated and untreated areas (χ2  =  129.81, df = 1, P  <  0.001). The resting density of females in human dwellings was three times higher in the unsprayed control areas (0.875 females per room (F/R) compared to the sprayed areas (0.318 F/R). The resting densities were comparable (χ2  =  0.014391, df = 1, P  =  0.9045) between villages sprayed once and villages sprayed twice.

### Molecular identification of the *An. gambiae* complex

Molecular identification of the 684 *An*. *gambiae* s.l. (442 by HLC and 242 by PSC) revealed the presence of *Anopheles arabiensis*, *Anopheles coluzzii*, *An. gambiae* sensu stricto (hereafter *An. gambiae*) and *Anopheles melas*. *Anopheles arabiensis* was the most frequent species of the complex, forming 75.56% (334/442) of biting and 82.6% (200/242) of resting populations captured. *Anopheles gambiae* was the least frequent species (3/684) and was collected only in the unsprayed control village of Keur Martin, where it was sympatric with *An. coluzzii* (70/684) and *An*. *melas* (77/684). Notably, *An. melas* was present only in Keur Martin.

Across the study area, *An*. *arabiensis* was found in significantly (χ2  =  136.63, df = 1, P  <  0.001) higher numbers in the unsprayed areas (363 captured in the unsprayed areas compared to 171 in the sprayed areas). However, its proportions were significantly lower in villages with only one spray round (12%; 63/534) than in those sprayed twice (20%; 108/534) (χ2  =  13.48, df = 1, P  <  0.001). While *An. arabiensis* and *An*. *coluzzii* were found in all study sites, including treated and untreated villages, both *An. melas* and *An*. *gambiae* were notably absent in the treated areas (Tables 3).

**Table 3:** Distribution of *Anopheles gambiae* complex species by villages and sampling methods

	*An. arabiensis*	*An. coluzzii*	*An. gambiae*	*An. melas*	
Control					
Gate Diocoul 2					
HLC	0	1	0	0	
PSC	0	0	0	0	
Keur Martin					
HLC	265	33	1	65	
PSC	98	28	2	12	
Sprayed once					
Takhoum Ndoundour					
HLC	4	1	0	0	
PSC	0	0	0	0	
Keur Massouka					
HLC	7	1	0	0	
PSC	52	0	0	0	
Sprayed twice					
Toucar centre					
HLC	8	4	0	0	
PSC	30	0	0	0	
Djilakh					
HLC	50	2	0	0	
PSC	20	0	0	0	
Total	534	70	3	77	684

*HLC* human landing catch, *PSC* pyrethrum spray catch

### Anthropophilic and parity rates

Of the 169 blood fed females (77 and 92, respectively from the treated and control areas) tested against the five potential vertebrate hosts (human, bovine, ovine, horse and chicken), 240 blood meals were successfully identified consisting of 105 simple meals and 135 mixed meals (with 114 and 21 comprised of blood from two and three different hosts, respectively) (Table 4). The proportions of human blood meals were 57.69% (75/130), in the unsprayed control areas, 30.77% (16/52) in the areas sprayed once, and 51.72% (30/58) in the areas sprayed twice.

**Table 4:** Proportion of simple and mixed blood meals by zone and host

	Identified (N)	Vertebrate hosts (%)					
		Human	Bovine	Ovine	Equine	Chicken	Mixed
Control							
Gate Diocoul 2	0	–	–	–	–	–	–
Keur Martin	92	47.83	0	3.26	11.96	0	36.96
Sprayed once							
Takhoum Ndoundour	0	–	–	–	–	–	–
Keur Massouka	40	22.5	0	2.5	47.5	0	27.5
Sprayed twice							
Toucar centre	20	20	0	5	5	0	70
Djilakh	17	70.59	0	0	0	0	29.41

However, vector populations’ preference for the human host was significantly higher in the unsprayed control areas compared to the areas sprayed once (χ2  =  9.7192, df = 1, P  =  0.001823), but comparable between the unsprayed area versus the area sprayed twice (χ2  =  0.362, df = 1, P = 0.55) (Table 4). The difference of the human blood indices was at the limit of significance between the two sprayed areas (χ2  = 4.1245, df = 1, P = 0.04), vector populations being slightly more anthropophilic in the area with two rounds of IRS compared to the other area sprayed once.

In the unsprayed control village of Keur Martin, *An*. *arabiensis* and *An*. *melas* displayed the same preference (Fisher’s exact test: P = 1; OR 0.694; CI 0.396–1.213) for human blood, with indices of 86.2% (50/58) and 81.82% (9/11), respectively.

Overall, the human blood index of *An*. *arabiensis* was significantly lower (Fisher’s exact test: P = 0.1831; OR 0. 694; CI 0. 396: 1.213) in the sprayed areas (59.7%; 46/77) than in the unsprayed control areas (86.2%; 50/58).

The ovaries of 213 females of *An. gambiae* s.l. were dissected for the vector populations age grading. The parity rate was significantly different between the two areas (Table 5); and was significantly higher (χ2  =  4.9711, df = 1, P  =  0.026) in the unsprayed control areas (64.5%; 113/175) than in the sprayed ones (43.2%; 16/37).

**Table 5:** Seasonal variation of Parity Rate of aggressive females

Month-year	Control						Sprayed once						Sprayed twice					
	Gate Diocoul 2			Keur Martin			Takhoum Ndoundour			Keur Massouka			Toucar centre			Djilakh		
	Dissected	Parous	P (%)	Dissected	Parous	P (%)	Dissected	Parous	P (%)	Dissected	Parous	P (%)	Dissected	Parous	P (%)	Dissected	Parous	P (%)
Oct-2013	0	0	0	29	15	52	1	0	0	0	0	0	1	1	100	8	5	63
Dec-2013	0	0	0	31	19	61	0	0	0	0	0	0	3	1	33	0	0	0
Mar-2014	0	0	0	1	1	100	0	0	0	0	0	0	0	0	0	0	0	0
Sep-2014	0	0	0	69	50	72	1	0	0	3	0	0	0	0	0	13	6	46
Oct-2014	1	0	0	44	27	61	1	1	100	0	0	0	1	0	0	4	1	25
Dec-2014	0	0	0	1	1	100	0	0	0	0	0	0	0	0	0	0	0	0
Mar-2015	0	0	0	0	0	0	0	0	0	0	0	0	0	0	0	0	0	0
Apr-2015	0	0	0	0	0	0	1	1	100	0	0	0	0	0	0	0	0	
Total	1	0	0	175	113	64.6	4	2	50	3	0	0	5	2	40	25	12	48

*P* Parity rate

### Circumsporozoite protein (CSP) and entomological inoculation rates (EIR)

The vector infection rates, evaluated from the HLC collection, were comparable (Fisher’s exact test: P = 1; OR 1.234; CI 0.154: 56.480) between the unsprayed control areas (1.9%; 7/365) and the areas sprayed twice (1.56%; 1/64). In the unsprayed areas, the infection rates were 1.1% (3/265) for *An. arabiensis*, 4.6% (3/65) for *An*. *melas* and 3% (1/33) for *An*. *coluzzii*. Only one female, of the species *An*. *coluzzii,* was found harbouring the *P. falciparum* CS protein in the sprayed areas. None of the 13 females tested in the areas sprayed once were infected (Table 6).

**Table 6:** Local and seasonal variation in infection rates of aggressive females of *An. gambiae* s.l.

Month-year	Control						Sprayed once						Sprayed twice					
	Gate Diocoul 2			Keur Martin			Takhoum Ndoundour			Keur Massouka			Toucar centre			Djilakh		
	Tested	Positive	CSPR (%)	Tested	Positive	CSPR (%)	Tested	Positive	CSPR (%)	Tested	Positive	CSPR (%)	Tested	Positive	CSPR (%)	Tested	Positive	CSPR (%)
Oct-2013	–	–	–	32	0	–	1	0	0	–	–	–	4	0	0	13	0	0
Dec-2013	–	–	–	72	0	–	–	–	–	–	–	–	5	0	0	–	–	–
Mar-2014	–	–	–	1	0	–	–	–	–	–	–	–	–	–	–	–	–	–
Sep-2014	–	–	–	165	2	1.2	1	0	0	7	0	0	2	0	0	35	0	0
Oct-2014	1	0	0	92	3	3.2	2	0	0	–	–	–	1	1	100	4	0	0
Dec-2014	–	–	–	2	2	100	–	–	–	–	–	–	–	–	–	–	–	–
Mar-2015	–	–	–	–	–	–	–	–	–	–	–	–	–	–	–	–	–	–
Apr-2015	–	–	–	–	–	–	1	0	0	1	0	0	–	–	–	–	–	–
Total	1	0	0	364	7	1.9	5	0	0	8	0	0	12	1	8	52	0	0

*CSPR* circumsporozoite rate

The entomological inoculation rates (EIRs) were significantly different between the sprayed and unsprayed areas. During the study period of 2 years, EIR was about 14 times higher in the unsprayed control areas than in the sprayed areas (13.14 infective bites per person, compared to 0.9). An unexplained relative increase (from 0 to 3.5 infective bites) of the EIR was noted in the sprayed zone, the second year after IRS implementation. This was possibly related to the natural inter-annual variation in malaria transmission.

Across the unsprayed control areas, malaria transmission occurred via three of the four *An. gambiae* complex members described in Senegal; *An. arabiensis*, *An*. *coluzzii* and *An*. *melas,* while in the sprayed areas only *An*. *coluzzii* was involved in the transmission.

## Discussion

During the study period, only four of the 21 anopheline species present in Senegal were identified [11, 12]: *An. arabiensis, An. melas, An. gambiae* and *An. coluzzi*, three of which are the main vectors of malaria across the African continent [13–16].

The sympatric existence of *An. melas* with *An. arabiensis* and *An. gambiae* in the study area can be explained by the presence of brackish water, the preferred breeding site of this species, as previously reported in the Saloum Delta [17, 18]. Among the collected species, *An. arabiensis* was the most frequent and widespread in the study area and is predominant across the country, except in the southern and south-eastern part of the country [19]. Notably, *An. arabiensis* accounts for the vast majority of anopheline in the Cape Verde Peninsula, in Pout and the Niayes area [20, 21], in the Senegal river Delta [22] in Barkedji [23], and in the northern and central regions of the country. The widespread nature of this species is explained by its better adaptation to drought and arid environment [24].

The results showed a clear impact of the IRS using the CS formulation of the pirimiphos-methyl (organophosphate). The anopheline fauna was significantly less abundant in the sprayed hot spots compared to the unsprayed control area. During the transmission period, the human biting rate of *An. gambiae* s.l. females was significantly lower in the sprayed hot spots than in the unsprayed control villages, and the EIR was around 14 times lower.

Surprisingly, *An. arabiensis*, the most common species during this study, was less frequent in areas with one spray round, compared to those sprayed twice. This could be attributed to the spatial environmental heterogeneity which may be suitable for vector proliferation in the latter areas than in the previous one. In addition, the known behavioural plasticity of *An. arabiensis* may be involved in the areas sprayed twice, allowing it to escape control interventions and maintaining higher population densities compared to areas with one spray round. Indeed, Gatton et al. [25] in their review on emerging and historical data on behavioural resistance in response to LLINs and IRS, reported that behavioural and species changes may be emerging, and that a preliminary model has demonstrated that behavioural resistance could have significant impacts on the effectiveness of malaria control. For instance, the use of insecticide may induce behavioural change in vector populations. However, the design of this study make difficult both data analysis and conclusion drawing on such phenotypical changes. Therefore, additional studies on the ecological, ethological and genetical characteristics of vectors populations are needed to better characterize the entomological impact of indoor residual spraying with pirimiphos-methyl in the residual transmission areas of Senegal.

Most of the biting occurred outdoors in both sprayed and unsprayed control areas. However, vector populations were slightly more aggressive outdoors in the former area. This might be explained by the predominance of *An. arabiensis* in the study area, which is known to be more exophilic than other species [26]. Nevertheless, the high coverage of pyrethroid-impregnated nets in both areas suggested an additional pressure towards outdoors biting behaviour due to the repellent effect of this class of insecticide, as shown previously in an experimental study [27]. In Benin, several studies have already shown that the IRS could have a dramatic decrease of the endophily rate [28].

Across the study area, half of blood meals were mixed including at least two vertebrate hosts with the human host in most of the cases. The human blood indices were moderate, being slightly higher in the unsprayed control areas compared to the two sprayed areas. This suggests vector populations’ inability to ensure a complete meal on the human host and their deviation to an alternative animal host to complete their meal. This could also be attributable to the highly zoophilic rate [26] of the main vector species, *An. arabiensis*, also known as a naturally evasive species due its behavioural avoidance of indoor insecticide exposure [29].

Despite the similar parity and infection rates between the unsprayed control areas and those sprayed twice, the overall longevity and entomological inoculation rates of vectors populations in the treated areas were markedly reduced. The behavioural plasticity of *An. arabiensis* allowing it to escape control interventions may also explain the relatively high parity rates and the subsequent higher EIR in the areas with two spray rounds. The low number of mosquitoes collected in the IRS areas doesn’t allows accurate comparison of the parity rates between the two areas. Therefore, additional studies are urgently needed to better understand the uncommon trends observed in this area. Despite the above limitation, the overall reduction of longevity and entomological inoculation rates in the treated areas could be attributed to the IRS interventions using a long-lasting insecticide formulation [30, 31]. This is supported by previous studies reporting significant reduction of the longevity of *An. gambiae* s.l. and *Anopheles funestus* across several IRS areas in Burundi [32, 33].

Overall, the results suggest IRS using the CS formulation of the pirimiphos-methyl (organophosphate) in hotspots is effective at reducing the risk of malaria transmission, despite anomalous results in the areas treated twice (which are probably due to the faculty of *An. arabiensis* to escape control interventions through its known behavioural plasticity [29]). Complementary studies are needed to better characterized *An. arabiensis* especially in residual transmission context to prevent it jeopardizing malaria elimination programmes. Longitudinal surveys are necessary to correctly assess the full impact of the IRS in the hot spots areas to better inform the best time of IRS Implementation owning potential monthly variation in transmission dynamics from year to year. In addition, considering the rainy season lasting from June to October, spraying activities should be implemented as earlier as possible, before mosquito populations peak, to maximize the full effect of IRS.

Despite giving some insights on the entomological impact of IRS with pirimiphos-methyl in the residual transmission areas, the lack of baseline data constitutes a major limitation in the study design which make it difficult to draw clear conclusions drawing. However, this limitation has been overcome with the inclusion of internal unsprayed control areas with the same ecological characteristics than the treated ones.

## Conclusion

The preliminary data from this pilot study showed that IRS with the CS formulation of pirimiphos-methyl is likely very effective in reducing residual malaria transmission risk in hot spots. If confirmed, this approach could be integrated into the nation-wide malaria control programme to target hot spot areas where malaria transmission is likely to become more frequent. However, additional studies including further longitudinal entomological surveys as well as ecological and ethological and genetical characterization of vectors species and their populations are needed to better characterize the entomological impact of indoor residual spraying with pirimiphos-methyl in the residual transmission areas of Senegal.

## Abbreviations

IRS: indoor residual spraying
SMC: seasonal malaria chemoprevention
HLC: human landing catch
PSC: pyrethrum spray catch
ELISA: enzyme-linked immunosorbent assay
HBR: human-biting rate
BPN: bites per person per night
IRD: indoor resting density
CSR: circumsporozoite rate
EIR: entomological inoculation rate
PCR: polymerase chain reaction
LLINs: long-lasting insecticide-treated nets
ITN: insecticide-treated net
NMCP: National Malaria Control Programme
PMI: President’s Malaria Initiative
WHO: World Health Organization
